# Distinction and Quantification of Noncovalent Dispersive and Hydrophobic Effects

**DOI:** 10.3390/molecules29071591

**Published:** 2024-04-02

**Authors:** Hans-Jörg Schneider

**Affiliations:** FR Organische Chemie, Universität des Saarlandes, D 66123 Saarbrücken, Germany; ch12hs@rz.uni-sb.de

**Keywords:** noncovalent interactions, dispersive and hydrophobic effects, measuring methods

## Abstract

The possibilities of comparing computational results of noncovalent interactions with experimental data are discussed, first with respect to intramolecular interactions. For these a variety of experimental data such as heats of formation, crystal sublimation heats, comparison with energy minimized structures, and spectroscopic data are available, but until now largely have not found widespread application. Early force field and QM/MP2 calculations have already shown that the sublimation heats of hydrocarbons can be predicted with an accuracy of ±1%. Intermolecular interactions in solution or the gas phase are always accompanied by difficult to compute entropic contributions, like all associations between molecules. Experimentally observed T∆S values contribute 10% to 80% of the total ∆G, depending on interaction mechanisms within the complexes, such as, e.g., hydrogen bonding and ion pairing. Free energies ∆G derived from equilibrium measurements in solution allow us to define binding increments ∆∆G, which are additive and transferable to a variety of supramolecular complexes. Data from more than 90 equilibrium measurements of porphyrin receptors in water indicate that small alkanes do not bind to the hydrophobic flat surfaces within a measuring limit of ∆G = ±0.5 kJ/mol, and that 20 functions bearing heteroatoms show associations by dispersive interactions with up to ∆G = 8 kJ/mol, roughly as a function of their polarizability. Aromatic systems display size-dependent affinities ∆G as a linear function of the number of π-electrons.

## 1. Introduction 

Noncovalent interactions determine a large part the chemistry of the new century. Many applications, such as for sensing/sensors, separations, drug design, pharmacophore, interactions in/with proteins and nucleic acids, protein design, medicinal diagnostics, noncovalent catalysis, and smart materials occur in solution, particularly in an aqueous environment. The interactions are often indiscriminately described as hydrophobic, although the most important contributions, namely dispersive interactions and hydrophobic factors, describe opposite mechanisms: Compounds lending themselves to lipophilic associations will prefer a hydrophobic environment, but interact less by London–type dispersive forces. These forces, customarily regarded as weak, have long been underestimated, due their small energies when compared to single noncovalent interactions and in solution due to the competition with a bulk solvent of sizable polarizability [1,2,3,4]. The essential role of polarizability both of the reaction partners and of the medium, as well as the large difference of host compounds’ propensity for dispersion interactions has been aptly reviewed [5]. That dispersion plays a role in solution is still disputed [6,7], but arguments such as that “…*vdWaals interactions are a simple function of molecular surface area*, *independent of atom type*” [8] contradict all experiments which do show large contributions by all kinds of heteroatoms [1]. For stacking in solution, dispersion is by no means a “*small component*” [9], and that intermolecular (stacking) interaction energies are largely attenuated or cancel out [10] is at variance with many measurements in water [1]. It has been argued that solvation and hydrophobic effects can also contribute to dispersive interactions [11]; this argument ignores that in water alkyl groups (Me, CHMe_2_, MeCH_2_ CH_2_) exhibit no measurable interactions with porphyrins [1]. In view of the widespread and promising applications, it is disturbing that the underlying fundamental mechanisms are still under dispute, and that computational predictions are not verified on a broader basis by experimental data.

## 2. Evaluation of Noncovalent Interactions and Benchmarking in Different States

Intermolecular interactions were at first correlated with the lattice energies and sublimation heats of crystals and the cohesive properties of liquids [12], then later in particular with measurements of the equilibria of many compounds. This most often used approach provides direct insight and numbers for the interaction energy of all kinds of noncovalent interactions [13,14,15,16,17,18,19,20,21,22]. Theoretical approaches [2,23,24] can help us understand the essential binding contributions of noncovalent interactions and to design new systems for manifold applications. The fundamental problem is the prediction of the free energy contributions ∆G, which determine the structures and properties of the molecular assemblies. Comparisons with experimentally known structures are most often applied as evidence to support theoretical predictions. This frequently used procedure of applying a theoretical prediction to a known structure often does show agreement, but does not necessarily determine the real energy minimum structure. To start with deliberately distorted conformers offers a more realistic way to arrive at the most stable structure and check the applied potentials.

In terms of most applications, the free energy ∆G of noncovalent interactions is the most important quantity relating to sensitivity and—within limitations—also to selectivity [25]. Corresponding benchmarks are available mainly from equilibrium measurements, also between conformers. Heats of formation frequently show good agreement between theory and experimental data, as can spectroscopic data. For crystals, sublimation heats directly measure the involved energies; hundreds of accurate data have been elaborated [26,27,28], but the data are barely used by computational chemists. For simple hydrocarbons, sublimation heats directly deliver the ∆H value for a single molecule. The experimental thermodynamic data as well as group contribution methods can be used to predict the thermodynamic quantities of organic compounds. The sublimation heats of compounds containing additional heteroatoms can also be evaluated, using atom-atom potentials and integral sums over the molecular electron density to obtain coulombic, polarization, dispersion, and repulsion lattice energies [29,30]. The possible failures of theoretical functions for noncovalent interactions have recently been outlined in detail [31].

## 3. London Dispersive Interactions

**Figure 1:**
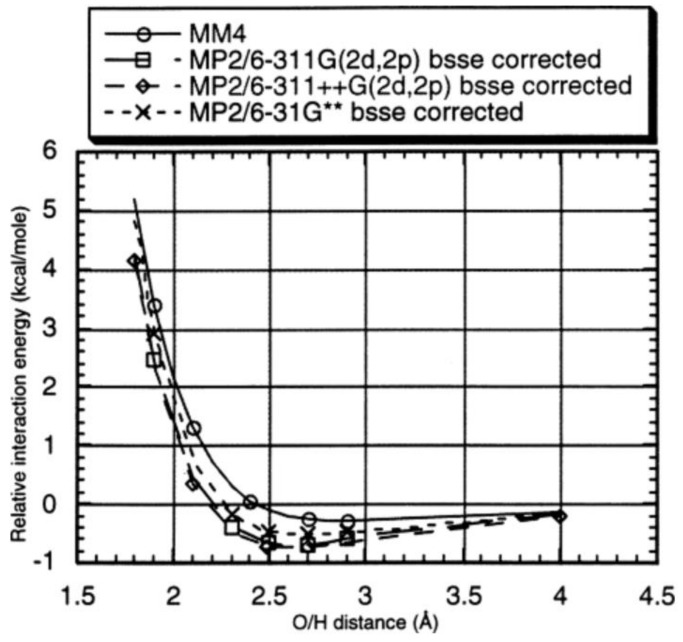
Calculated interaction energy potentials for the methane–dimethyl ether system. Allinger et al. [39]. Copyright 2000 American Chemical Society.

Cohesive solvent–solvent interactions are considered to be the major driving force behind apolar association in solution [32], for which reason water is a most effective and at the same time practically the most important medium. The importance of dispersive interactions has been recognized in many analyses [33,34,35,36,37], but still lacks to a large degree experimental verification. The first theoretical evaluations of dispersion interactions go back to N. L. Allinger [38,39], who in 1989 used Buckingham-type potentials (Figure 1) and a combination of force field (MM) and QM/MP2 calculations to demonstrate an agreement between experiment and theory with still-unsurpassed accuracy (Table 1). One of the few comparisons of sublimation heats arrived with the London-Eisenschitz equation, with semiempirical dispersion correction for the benzene dimer at the far-off value of 6.4 instead 10.4 kcal/mol [40].

Recently, a universal 1/R^−3^ decay instead the usual 1/R^−6^ has been proposed and confirmed with H-2 and He-2 molecules [41]. Other QM–derived potential functions have been described for van der Waals interactions, and have been tested by atomic force microscopy measurements [42]. A set of “semiexperimental” equilibrium geometries of noncovalent complexes were compared to ab initio data, with structures based on spectroscopic data combined with vibrational corrections at the double-hybrid density functional level; the obtained benchmark-quality data comprised 16 complexes including dispersion interactions [43]. Up to 89 different ab initio methods with dispersion correction have been tried as a means of predicting noncovalent bond length for comparison with data from microwave spectroscopy [44].

## 4. Association Energy between Molecules in Solution—Entropic Contributions as a Major Problem

**Figure 2:**
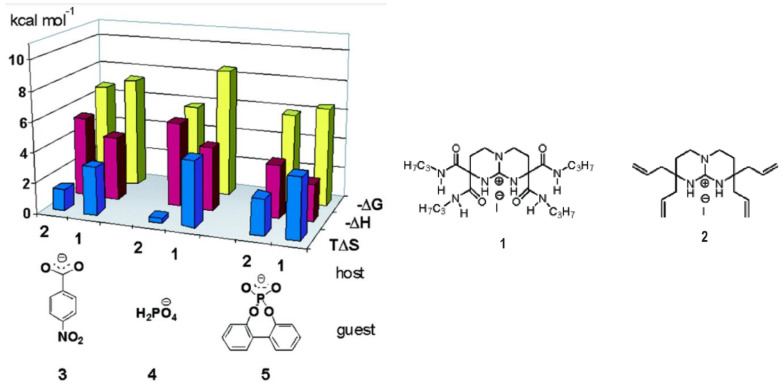
A bicyclic guanidinium anion receptor which binds oxoanions, preferably by hydrogen bonding (with **1**) or ion pairing (with **2**), resulting in smaller or larger entropic contributions Adapted with permission from Jadhav et al. [54]. Copyright 2005 American Chemical Society.

**Figure 3:**
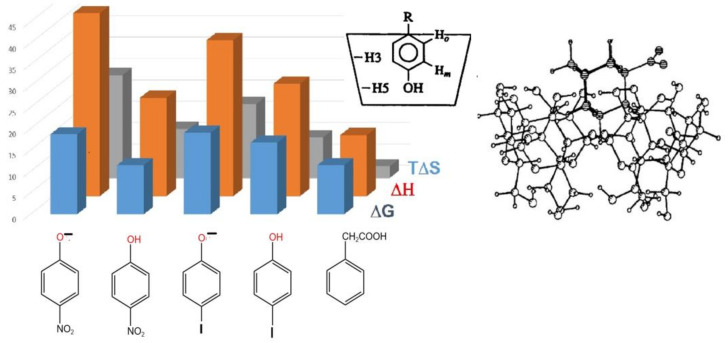
Large variation of thermodynamic parameters (in kJ/mol) from calorimetry for some α-cyclodextrin complexes where intracavity inclusion was secured by NMR spectroscopy. Data from V. Rüdiger et al. [55].

**Figure 4:**
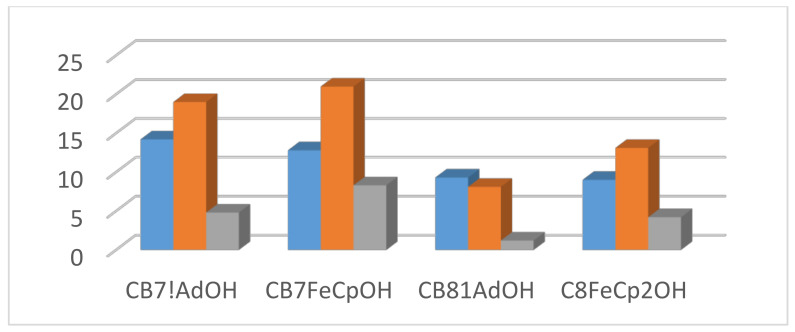
Typical variations of ∆G (blue), ∆H (red), and T∆S (green) values [all in kJ/mol] for cucurbit[*n*]uril complexes; data from Biedermann et al. [56].

**Figure 5:**
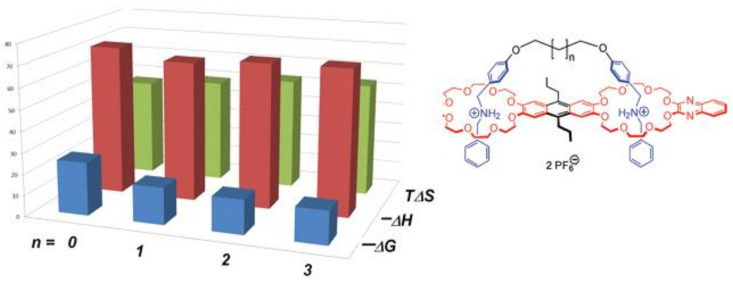
Similar magnitude of thermodynamic parameters (in kJ/mol) in crown-ammonium pseudorotaxane complexation; binding as a function of spacers of different length; data from Jiang et al. [48].

While the molecules and complexes dominated by noncovalent interactions so far discussed are essentially free from entropic contributions [45], the opposite holds true for associations between molecules in solution as well as in the gas phase. Alone, the loss of translatory freedom can theoretically reach 65 kJ/mol [46,47], a number dependent on standard concentration. With supramolecular complexes, typical values of T∆S = 5 to 9 kJ/mol are often observed [14,17,18], but values of 50 kJ/mol have been found [48]. Entropic contributions were also considered in theoretical analyses of noncovalent bound complexes [49,50], but were rarely compared to experimental data. The large and difficult to predict variations of thermodynamic parameters have been reviewed for complexation with, e.g., ionophores [51] or cyclodextrin [52]; typical examples are illustrated in Figure 2, Figure 3, Figure 4 and Figure 5.

Entropically reduced binding energies ∆G occur in many complexes, where beside ion pairing, hydrogen bonds and other interactions play a role [53]. The bicyclic guanidinium anion receptors in (Figure 2) with sec-carboxamido groups bind in acetonitrile, effectively oxoanions [54]. Receptor **1** offers a multitude of hydrogen bonds, and its affinity ∆G is still dominated by ∆H, while the interaction with receptor **2** occurs essentially by ion pairing, which is known to be essentially entropy-driven.

In addition, exceedingly large adverse entropic factors occur if rotational freedom is strongly restricted at several places as seen in Figure 5.

Data from many other complexes show that adverse T∆S values can determine the free binding energy ∆G in solution by 10% to up to 80%; only ion pairing in water is almost entirely entropy-driven [14,17,18]. Another complication for theoretical approaches is the often strong temperature dependence [57,58,59,60,61] of the thermodynamic parameters (Supplementary Material Figure S1).

In spite of the obvious limitations imposed on prediction of the binding strength ∆G by significant T∆S contributions, good agreement between theory and experiment has been claimed for many complex formations in solution [34,62]. The conversion of ∆G values measured in solution to, e.g., the gas phase values is often also problematic; COSMO continuum solvation models were used, for instance [63], which is particularly questionable for complexes which owe their often very high stability to the liberation of distorted cavity water and not to dispersive interactions, see Section 5 [64].

**Figure 6:**
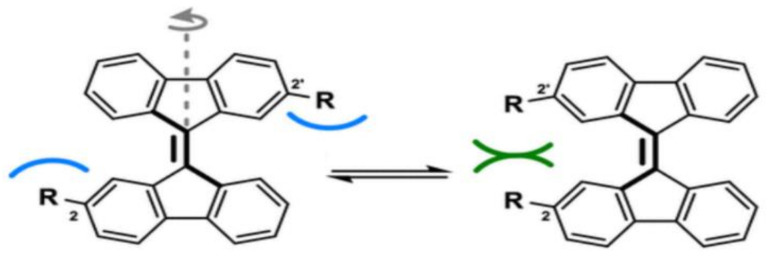
A bifluorenylidene balance with a z- preference for alkyl residues R.

Large entropic contributions explain why noncovalent interactions can be weak in solution but quite strong in solids and molecular balances [65,66,67], where the measured equilibria are essentially also free from entropic disadvantage. A bifluorenylidene balance exhibits a striking case where the contact between alkane residues R shifts the equilibrium to the *z*-site, for R = cyclohexyl with ∆G_Z/E_ = −2.5 kJ/mol in organic solvents [68] (Figure 6). Alkanes are characterized by a relatively smaller polarizability than compounds of the same size with heteroatoms, but strong intramolecular dispersive forces in crowded pure hydrocarbons lead to strong distortions, including, e.g., bond elongation [69]. A related affinity increase for binding carbohydrates was observed by introduction of, e.g., cycopentyl groups into the receptor cavity, due to additional van der Waals forces, but possibly also due to the preferred guest accommodation in a more lipophilic cavity [70].

Detailed studies on alkyl–alkyl interactions have been aptly reviewed in the context of supramolecular chemistry [71], most often with molecular balances which, although in solution, are essentially free from entropic contributions. Investigations with molecular balances indicate solvophobic effects are major forces for alkyl–alkyl aggregation in solution [65].

The strong intramolecular noncovalent interactions of hydrocarbons are in sharp contrast to their interactions in bimolecular associations. There they are negligible in comparison to compounds bearing π-groups or heteroatom, which exhibit significant affinities in measurements with flat porphyrin models [1]. The adverse entropic contributions T∆S (see Figure 3, Figure 4 and Figure 5) here are larger than the enthalpy gain ∆H by the few and weak interactions with the C-H-bonds and a receptor surface.

## 5. Consistent Experimental Free Energy Increments for Dispersive Interactions

It was shown that alkanes, in spite of their high hydrophobicity, exhibit no measurable association with flat aromatic surfaces like porphyrins, which eliminates hydrophobic factors as a driving force for all other compounds with larger hydrophilicity, e.g., those with heteroatoms [1].

**Figure 7:**
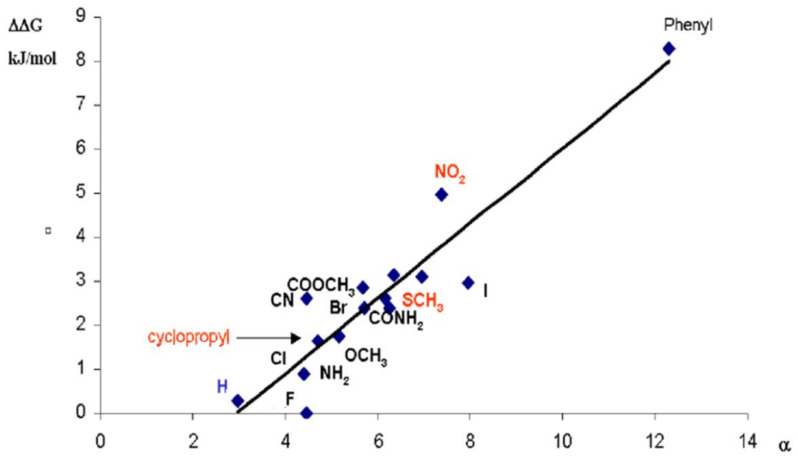
Free energy increments ΔΔG for the association between porphyrins and different groups X as function of molecular polarizabilities of MeX.

The absence of alkane association on π-surfaces allowed us to derive binding increments for all kinds of non-hydrocarbons; even introduction of the weakly polarizable fluorine leads to a measurable affinity [1]. This LFER-type approach, following in the footsteps of Louis Hammett, assembles a sufficiently large number of equilibrium constants on the basis of suitably designed complexes [14,17,18]. Their ∆G values must be additive, transferable, and exhibit linear correlation with the number of observables (Figure S2). The ∆G values exhibit a moderately linear correlation with the polarizability of the guest molecules, whereby the large number with phenyl (due to its large size) largely determines the slope of the correlation line (Figure 7).

The additivity of the dispersive ∆G values and their additional applicability to biopolymers is visible in associations with oligopeptides (Figure S4). Protein folding is also known to be accompanied by van der Waals interactions [72]. Recent simulations of peptide folding show partial control by such additional dispersive interactions [73]. In a peptide, amide cis–trans isomerism has been ascribed to a 60% vdW contribution [74].

## 6. Hydrophobic Effects

The “classical” hydrophobic effect is thought to be due to the release of solvation of “structured” water between solutes and bulk water, and it is characterized by a typical entropy increase [75,76]. Although it is widely accepted that the origin of a hydrophobic association is not the affinity between lipophilic solutes but the accompanying solvation changes, the detailed understanding of these processes has led to a multitude of mostly theoretical papers, which also include the possibility of enthalpy-driven hydrophobic associations [77,78,79,80,81,82,83,84,85,86,87]. The problem of the distinction and quantification of the noncovalent van der Waals interactions also arises with the many artificial supramolecular complexes studied in water [88,89,90,91] and can not yet be resolved. Complexations of alkanes with ß-cyclodextrin are indeed almost entirely entropy-driven, with small ∆H values close to those of the transfer from water to hydrocarbon, while those with the smaller α-cyclodextrin cavity are largely enthalpy-driven [92]. However, the assignment is ambiguous in view of the temperature dependence [60,61] of the thermodynamic parameters, whereby at higher temperatures enthalpy-driven complexation often changes to entropy-driven complexation. Negative heat capacity values (Δ*C*p) were also regarded as evidence for classical hydrophobic effects [92], but are again ambiguous as is visible in Δ*C*p values of similar magnitude: complexes of, e.g., a cyclophane with benzene derivatives with dominating dispersive interactions exhibits Δ*C*_p_ values from 84 to 250, whereas those with cyclodextrins with dominating hydrophobic effects range from 270 to 500. Solvent effects, which help to distinguish, e.g., ionic from dispersive interactions [93] are again similar for dominating hydrophobic (e.g., with cyclodextrin complexes [94]) and dispersive effects (e.g., between porphyrins and arenes [95]), with water as the most favorable medium in both cases.

The striking result from the comparison of complexes with alkane residues is that within the error of ±0.5 kJ/mol there is no interaction between the porphyrins π surface and alkanes [1], even though both represent a hydrophobic moiety. This is obvious from the ∆G values for cyclohexanoic and propionic acid, which showed only salt bridge interactions near the common values of ∆G_ip_ = (5 ± 1) kJ/mol, and the negligible contribution of alkyl substituents in benzoic acids with (with 4-Me, 4-CHMe_2_ and 3,5-di-Me). These results are the first experimental evidence for the absence of any hydrophobic contributions in associations between small flat particles.

The absence of hydrophobic associations at flat surfaces does not exclude those in cavities, which can be considered as nonclassical hydrophobic interactions [96]. The designation “nonclassical” has been reserved until now for associations which are enthalpy-driven, as observed early on with aromatic cyclophanes and aromatic guest molecules [97,98]. These complexes are, however, dominated by dispersive interactions.

Many receptors have confined cavities in which water is, other than in porphyrins complexes, in a disordered network. Replacement of such high-energy water molecules, which have less than four hydrogen bonds, contributes significantly to guest binding [99]. A recent investigation with cucurbit[*n*]uril complexations concluded that solvation free energy differences between the host–guest complexes and between the unbound host and guest must indeed play a “peculiar” role, and less satisfactory agreement was observed between the computed and experimental ∆G values [100]. Isothermal titration calorimetry also showed large variations between the thermodynamic signatures for these complexes. Explicit solvent molecular dynamics simulations for association with a concave surface also ascribed the expulsion of disorganized or high-energy water in a receptor pocket as enthalpy-driven binding [101]. Statistical–mechanical calculations indicate entropy-driven associations of small molecules, ellipsoids, and plates, with opposed entropy interactions for concave surfaces, and can explain the occurrence of both entropy-driven and -opposed hydrophobic effects [96]. In confined spaces, water can exert less interwater hydrogen bonds than in bulk water, where on the average close to four bonds materialize [102,103].

**Figure 8:**
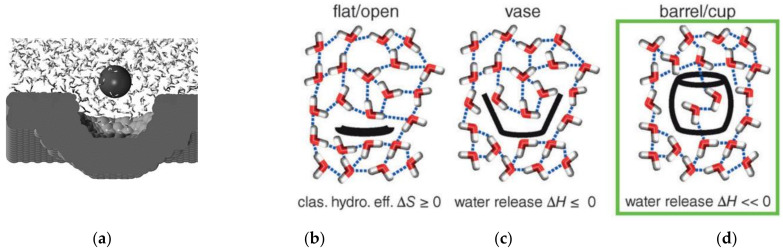
Intermolecular hydrogen bonds of water molecules at (**a**) concave; (**b**) flat, (**c**) curved cavity and (**d**) deeper inside receptors; (**b**–**d**) from Biedermann et al. [99]. 2014, Copyright John Wiley & Sons, Inc.

The release of high-energy water from cavities as a driving force for complexation was suggested early on for cyclodextrin complexes [104], water predicted by MD simulations for water in cyclophanes [105], and in the last few years put on a firm basis with extensive analyses of complexes with cucurbiturils (CBs) [3,106,107]. These barrel-shaped receptors have low polarizability inside the cavity and no hydrogen bond acceptors or donors inside; they contain only a few water molecules in their cavities, depending on the cavity size (Figure 8) [99]. These factors lead to extraordinarily high binding enthalpies of up to ∆H = 90 kJ/mol, larger than observed with any biological receptors [3], and to a small dependence on specific binding sites in comparison to many other supramolecular complexes. The binding strength of alkenes in CBs is, however, stronger relative to alkanes, and increases roughly with the size/polarizability of the hydrocarbons, which speaks to some dispersive contribution [108]. Another solvophobic contribution has been proposed for cavitation energies which, e.g., for noble gases are smaller in the cucurbit[5]uril cavity than that necessary for cavity creation in bulk water [109].

**Figure 9:**
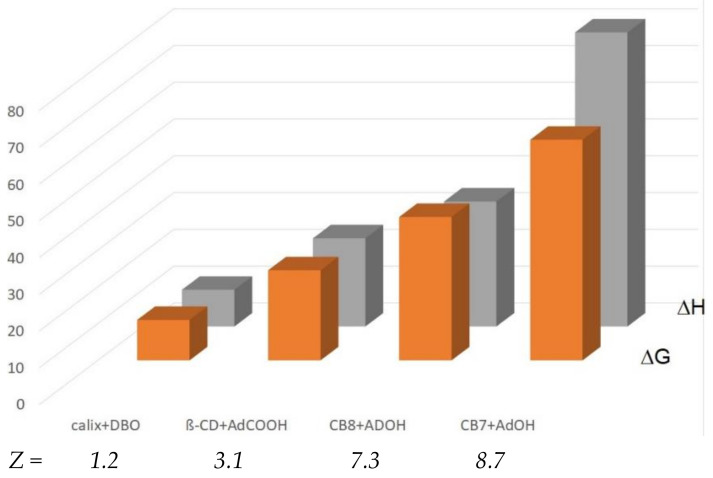
The increase of ∆G and ∆H (in kJ/mol) with the high-energy water efficiency number *Z*.

Water with less than the optimal number of hydrogen bonds occurs in many supramolecular receptors, including CBs, cyclophanes, cryptophanes, pillarenes, fullerenes, and calixarenes [3]. As a measure of the high-energy water contribution, one can use a factor *Z* = *N* (3.62 − *m*), in which *N* is total number of water molecules in a cavity and *m* = 3.62 is the optimal number of interwater hydrogen bonds in bulk water. (The value of *m* = 3.62 instead of *m* = 4 was used in the water box simulations in order to bring the data on scale) [99]. Figure 9 illustrates how both ∆G and ∆H increase with the efficiency number *Z*, with rather small entropic differences, with guest molecules which exert no or small additional noncovalent interactions inside the cavity.

## 7. Conclusions

The experimentally-secured distinction between different hydrophobic and dispersive interactions and the available energy increments for dispersive interactions call for a reconsideration of traditional approaches, which for hydrophobic contributions were until now based on, e.g., the evaluation of contact surfaces, or the use of parameters derived from partition coefficients [110]. Classical hydrophobic interactions between small flat or ellipsoid [111,112] particles do not exist in reality. Nonclassical hydrophobic interactions can be large in cavities and depend on the amount of disorganized or high-energy water molecules in the cavities. Their contributions can be estimated with the number *Z*, which reflects the number of distorted water molecules and their hydrogen bond-deficiency. In view of their similar thermodynamic signature and solvent dependence, dispersive interactions are often considered as hydrophobic. This is particularly questionable with biopolymers [113], where many amide, amino, hydroxy, thio, and aromatic groups lend themselves to dispersive interactions. Dispersive interaction energies can be quite sizable, with for example between an aromatic surface and a phenyl ring of about 7 kJ/mol, or those with, e.g., a tetrapeptide (gly4) 7 kJ/mol. It has been pointed out that the surface of proteins is nonhomogeneous with respect to hydrophobicity, roughness, and topology [114], which may also leave room for nonclassical hydrophobic interactions. The large and variable entropic contributions with associations in solution require explicit T∆S calculations; theoretically evaluated ∆H values could in the future at least be compared to experimental values measured by calorimetry. Platforms such as porphyrins offer themselves for testing the dispersive interactions of parts of biopolymers or of drugs.

## Figures and Tables

**Table 1:** Calculated and experimental sublimation heats (kcal/mol) for four hydrocarbons; data from Lii et al. [38].

	MM2	MM3	Exp. *
C_6_H_6_	11.18	10.32	10.42
C_6_Me_6_	24.81	16.83	17.86
n-hexane	19.31	11.59	9.76
n-dodecane	38.77	24.52	23.78

## Data Availability

Not applicable.

## Supplementary Materials

The following supporting information can be downloaded at: https://www.mdpi.com/article/10.3390/molecules29071591/s1. Figure S1. Temperature dependence of thermodynamic parameters in associations. Figure S2. Additivity: Prediction of ∆G of 50 different porphyrin complexes with increments for 12 increments for different functionalities. Figure S3. Affinities (∆G in kJ/mol) of halogen derivatives. Figure S4. Association of peptides with the porphyrins TppyP. Figure S5. Stacking interactions with arenes: correlation with number of π-electrons. Reference [115] is cited in the Supplementary Materials.
