# Supplementary material for: Distinction and Quantification of Noncovalent Dispersive and Hydrophobic Effects
Source: Molecules. 2024 Apr 2;29(7):1591. doi: 10.3390/molecules29071591 (PMC11013637; doi:10.3390/molecules29071591)
Supplement: Supplementary file 1 [file molecules-29-01591-s001.zip › molecules-2928135-supplementary.pdf]

Supplementary Material

To Hans-Jörg Schneider

Distinction and quantification of noncovalent dispersive and hydrophobic effects

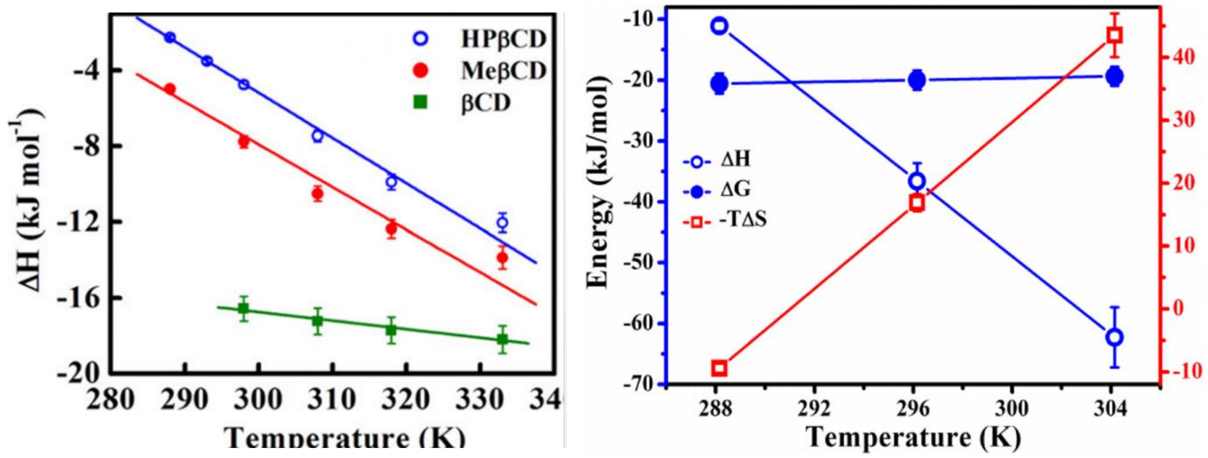

(a) (b)

Figure S1. Temperature dependence of thermodynamic parameters in associations (a) enthalpy ΔH with sodium glycocholate and cyclodextrins as host (βCD, (2-hydroxypropyl)-βCD **HPßCD**, and methyl βCD **MeßCD**); (b) interaction of the indoloquinoline alkaloid cryptolepine with natural RNA duplex . Reprinted with permission by the American Chemical Society from a) Paul, B.K., Ghosh,N. Mukherjee, S. *J. Phys. Chem. B* 2016, 120, 3963−3968: b) Nandy,A., Shekhar,S. , Paul, B.K., Mukherjee, S. *Langmuir* 2021, 37, 11176−11187

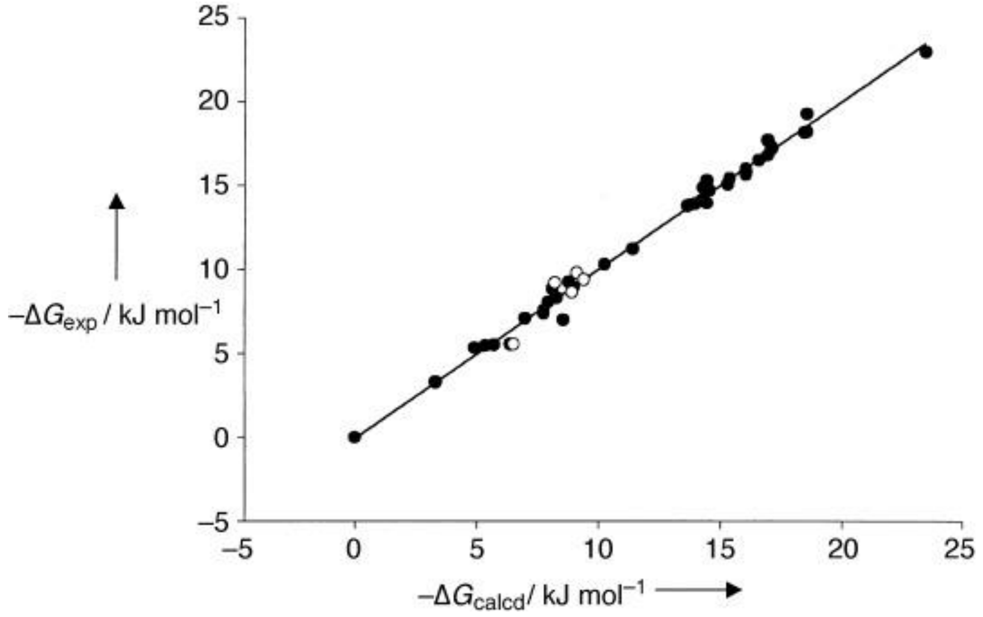

Figure S2. Additivity: Prediction of ΔG of 50 different porphyrin complexes with increments for 12 increments for different functionalities

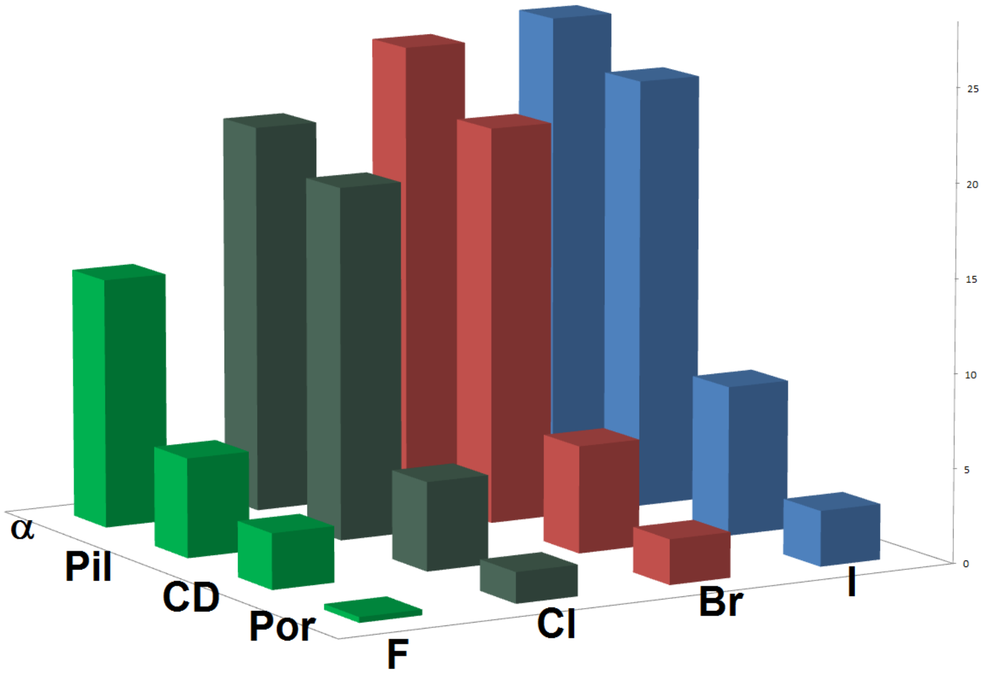

Figure S3. **Affinities** (DG in kJ/mol) **of halogen derivatives**

with porphyrins (**Por**, DDG increments)

with a-cyclodextrin (**CD**)

with pillarene (**Pil**)

and polarizabilities **a** (relative values) of corresponding compounds MeR

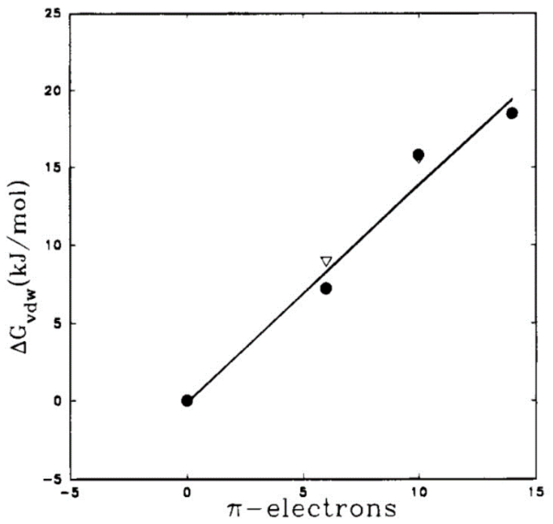

Figure S4. Stacking interactions with arenes: correlation with number of π -electrons

Schneider, H.-J.; Wang, M.: "Ligand-Porphyrin Complexes: Quantitative Evaluation of Stacking and Ionic Contributions[1]" *J. Org. Chem.*, **1994**, *59*, 7464-7472

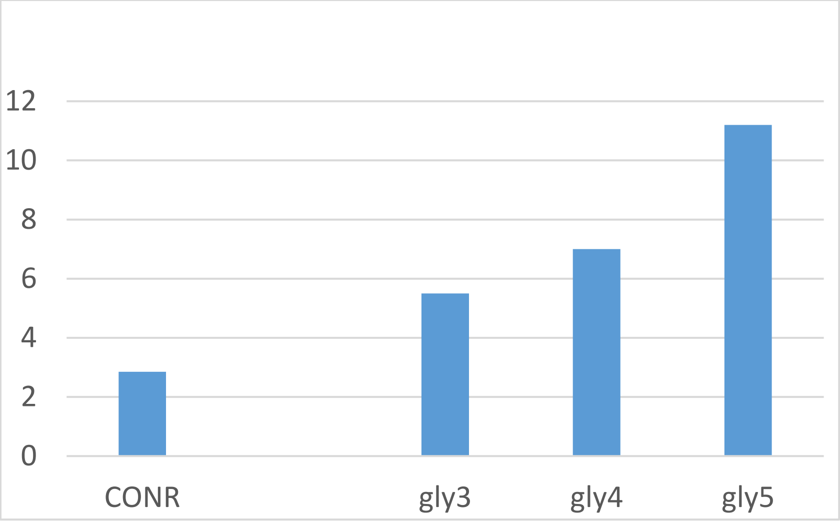

Figure S5. Association of peptides with the porphyrins TPpyP , ΔG in [kJ/mol], gly-gly not measured.
